# The Synthesis, Properties, and Stability of Lithium-Containing Nanostructured Nickel-Doped Ceramics

**DOI:** 10.3390/gels8070451

**Published:** 2022-07-19

**Authors:** Artem L. Kozlovskiy, Maxim V. Zdorovets, Ainagul A. Khametova, Dmitriy I. Shlimas

**Affiliations:** 1 Laboratory of Solid State Physics, The Institute of Nuclear Physics, Almaty 050032, Kazakhstan; mzdorovets@gmail.com (M.V.Z.); shlimas@mail.ru (D.I.S.); 2 Engineering Profile Laboratory, L.N. Gumilyov Eurasian National University, Nur-Sultan 010008, Kazakhstan; khametovaaa@gmail.com

**Keywords:** lithium-containing ceramics, blanket, doping effect, resistance to mechanical stress, hardness

## Abstract

Lithium-containing ceramics have several great potential uses for tritium production, as well as its accumulation. However, their use is limited due to their poor resistance to external influences, mechanical pressure, and temperature changes. In this work, initial nanostructured ceramic powders were obtained using the sol-gel method, by mixing TiO_2_ and LiClO_4_·3H_2_O with the subsequent addition of NiO nanoparticles to the reaction mixture; these powders were subsequently subjected to thermal annealing at a temperature of 1000 °C for 10 h. Thermal annealing was used to initiate the phase transformation processes, and to remove structural distortions resulting from synthesis. During the study, it was found that the addition of NiO nanoparticles leads to the formation of solid solutions by a type of Li_0.94_Ni_1.04_Ti_2.67_O_7_ substitution, which leads to an increase in the crystallinity and structural ordering degree. At the same time, the grain sizes of the synthesized ceramics change their shape from rhomboid to spherical. During analysis of the strength characteristics, it was found that the formation of Li_0.94_Ni_1.04_Ti_2.67_O_7_ in the structure leads to an increase in hardness and crack resistance; this change is associated with dislocation. When analyzing changes in resistance to cracking, it was found that, during the formation of the Li_0.94_Ni_1.04_Ti_2.67_O_7_ phase in the structure and the subsequent displacement of the Li_2_TiO_3_ phase from the composition, the crack resistance increases by 15% and 37%, respectively, which indicates an increase in the resistance of ceramics to cracking and the formation of microcracks under external influences. This hardening and the reinforcing effect are associated with the replacement of lithium ions by nickel ions in the crystal lattice structure.

## 1. Introduction

One of the most promising and urgent issues in the development of thermonuclear energy is the issue of tritium accumulation. Tritium is one of the key elements for maintaining thermonuclear reactions and, as a result, for the performance of thermonuclear reactors [1,2]. The design of the ITER reactor requires a sufficiently large amount of tritium to maintain operation, and the currently known technologies for its production cannot entirely fulfil this demand. In this regard, for several years, much attention has been paid to the search for alternative ways of producing tritium, as well as the possibilities of its accumulation for further exploitation. At the same time, interest in tritium fuel is quite large not only due to its energy yield, but also due to the absence of long-lived decay products, as in the case of uranium or plutonium fuel, which require long-term storage [3,4,5]. 

The most promising direction in this area of research is the creation of blanket or breeder structures, consisting of lithium-containing ceramics, which are capable of producing and accumulating tritium under the action of neutron radiation and subsequent neutron-initiated nuclear reactions of the (n, Li) → (T, He) type [1,4]. 

Lithium-containing ceramics are chosen as the main material for breeding tritium due to the possibility of nuclear reactions occurring in them with the subsequent formation of tritium, and because of the high concentration of lithium in nature, which would solve the issue of tritium production for the next few decades. As a rule, structures of the Li_2_TiO_3_, Li_2_SiO_3_, and Li_2_ZrO_3_ types are chosen as lithium-containing ceramics; these structures have a combination of physicochemical and structural properties that make it possible to use them for tritium production [6,7,8,9,10]. At the same time, one of the key requirements for lithium-containing ceramics based on titanates and silicates is an increased resistance to mechanical and radiation damage in the case of their operation in the production of tritium [11,12,13,14,15]. To this end, in the past few years, various options for modification or doping have been proposed to increase stability and productivity, as well as to preserve the materials’ structural and strength properties during production of tritium and its absorption and desorption [16,17,18,19,20,21,22].

One of the options for modifying lithium-containing ceramics based on titanates is doping them with nickel or its oxide to obtain complex phases in the structure of ceramics [23,24,25]. Nickel is chosen as a dopant due to its strong ability to absorb neutron radiation, as well as the possibility of increasing the strength properties of the materials. At the same time, as a rule, such research pays much attention to the study of structural and phase transformations during doping, as well as the influence of the dopant and phase composition on the strength properties of ceramics, which play a significant role in determining their operation efficiency and scope [23,24,25].

On this basis, the purpose of this work is to establish the relationship between changes in the structural and strength properties of lithium-containing ceramics, as well as to assess their resistance to external influences in the case of doping them with nickel oxide; nickel oxide is chosen due to its properties when absorbing ionizing radiation, including neutron radiation. The relevance of this study lies in the possibility of increasing the resistance of lithium-containing ceramics based on titanates to mechanical and radiation damage, which can lead to the destabilization of the structural and strength properties of ceramics. 

## 2. Experimental Section

For the synthesis of lithium-containing ceramics doped with nickel, the following chemical reagents were used: TiO_2_, LiClO_4_·3H_2_O, and NiO nanoparticles from Sigma Aldrich (Sigma Aldrich, USA). The chemical purity was 99.95%. Synthesis of lithium-containing ceramics was carried out using the sol-gel method [11,12], by the weighing and subsequent dissolution of TiO_2_ and LiClO_4_·3H_2_O reagents, followed by the addition of NiO nanoparticles to the reaction mixture. After synthesis, the resulting mixture was annealed in a muffle furnace at a temperature of 1000 °C for 10 h, followed which the resulting powder was cooled, together with the furnace, for 24 h. The stoichiometric ratio of the TiO_2_ and LiClO_4_·3H_2_O components in the reaction mixture was 1:1. The doping of NiO with nanoparticles to obtain ceramics containing substitutional or interstitial solid solution phases was carried out by adding nanoparticles to the resulting reaction mixture in a molar ratio of 0.10 and 0.25 mol.

The analysis of morphological features, including the determination of the geometry and sizes of grains that make up the synthesized ceramics, was carried out using the method of scanning electron microscopy, implemented on a Hitachi TM 3030 (Hitachi, Tokyo, Japan) scanning electron microscope with the possibility of mapping and energy dispersive analysis. Mapping was performed to determine the uniformity and isotropy of the nickel dopant distribution in the structure of the synthesized ceramics.

Analysis of the structural characteristics, as well as of the change in the phase composition of ceramics depending on the concentration of the NiO dopant, was carried out using the X-ray diffraction method. The diffraction patterns were taken on a D8 Advance ECO powder diffractometer (Bruker, Karlsruhe, Germany), using Bragg–Brentano geometry in the angular range of 2θ = 20–80°. Analysis of the phase composition was undertaken by comparing the diffraction lines with the results of the ICCD database.

For mechanical tests, the synthesized ceramics were pressed in special molds, which made it possible to create cylindrical tablets 5 mm in diameter and 1 mm thick. Determination of the effect of dopant concentration on resistance to mechanical stress, including compression and crack resistance, was carried out according to standard methods for approved indentation, as well as single compression. The hardness value was determined by the indentation method using a Vickers pyramid as an indenter at an indenter load of 100 N. The single compression method was used to determine the crack resistance of the synthesized ceramics. This method consists of a single compression of the studied samples at a constant compression rate of 0.2 mm/s, which makes it possible to determine the maximum pressure that ceramics can withstand before they crack. 

## 3. Results and Discussions

**Figure 1:**
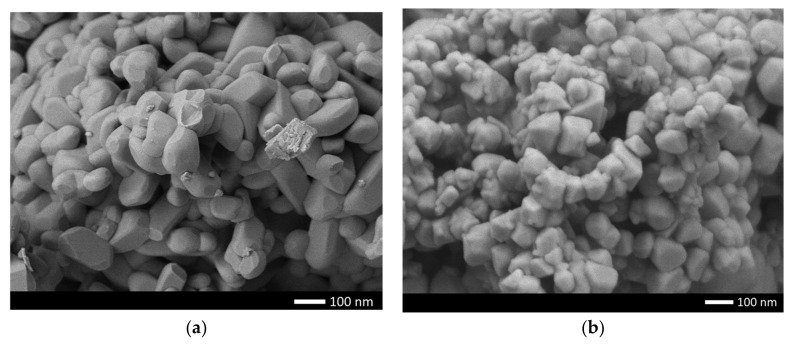

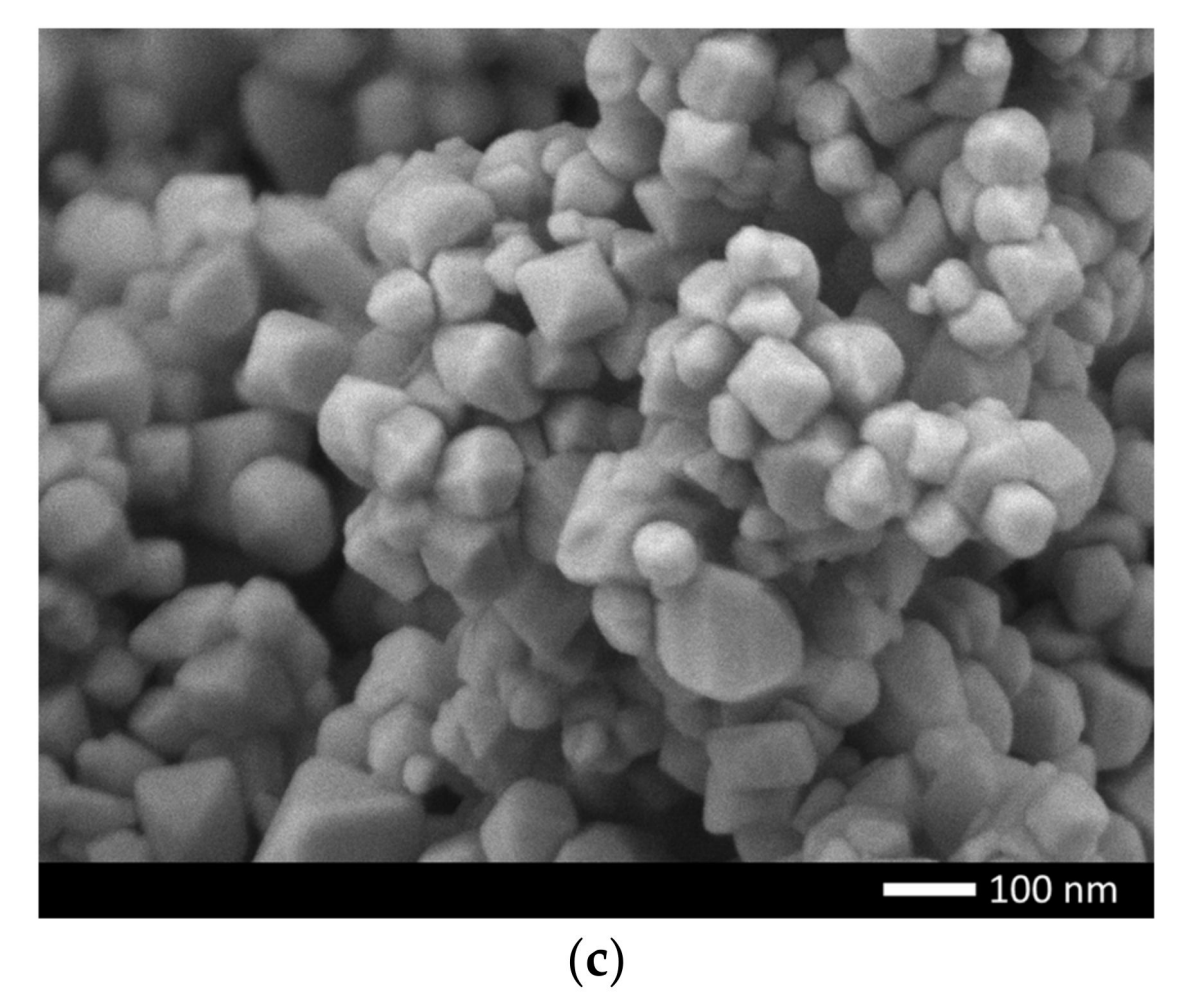
SEM images of the morphology of the synthesized ceramics: (**a**) pristine; (**b**) 0.10 mol; (**c**) 0.25 mol.

Figure 1 shows the results of measuring the morphological features of the synthesized ceramics depending on the dopant concentration. As can be seen from the data presented, in the initial state the average grain size is 70–80 nm, while the shape of the grains is close to ellipsoidal or spherical. Doping of NiO with nanoparticles leads to the formation of smaller grains, as well as a change in their shape from ellipsoid to cubic or rhomboid. Such a change in the shape and size of grains is because of a change in the phase composition of ceramics associated with the processes of phase transformations because of doping. At the same time, a change in the grain size leads to the formation of dendrite-like formations, with many grain boundaries and joints.

**Figure 2:**
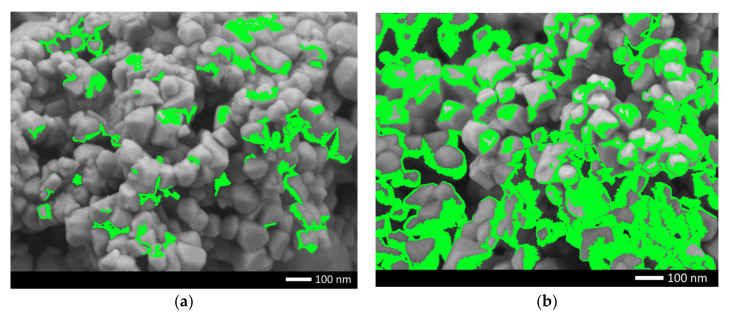
Mapping results reflecting nickel distributions in the structure of ceramics after doping: (**a**) 0.10 mol; (**b**) 0.25 mol. (Green reflects the distribution of nickel in the structure of ceramics).

To determine the isotropy of the dopant distribution in the structure of the synthesized ceramics, the mapping method was used, the results of which are shown in Figure 2. In the case of a dopant concentration of 0.10 mol, according to the maps of the distribution of elements, the nickel content is uneven in the structure, which indicates that there are two types of ceramics in the structure particles; in some of these, the nickel content is sufficient for its identification. At a dopant concentration of 0.25 mol in the composition of ceramics, according to the element distribution maps, the nickel content in the grain structure is isotropic, which indicates that phase transformation processes are observed in the ceramic structure, with an increase in the dopant concentration due to the substitution or interstitial effect. 

**Figure 3:**
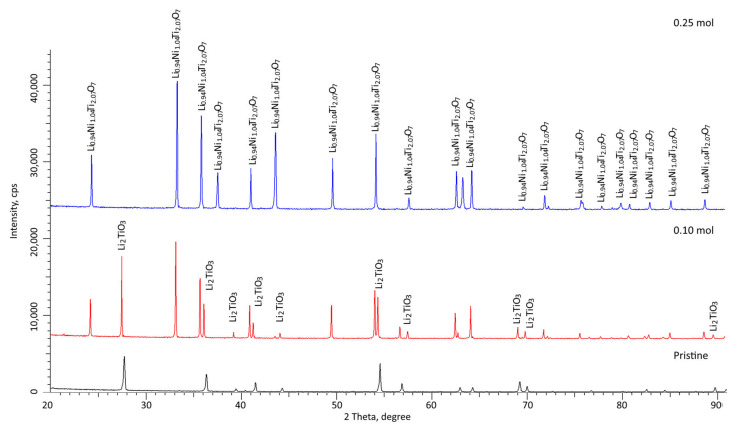
The results of X-ray diffraction of the studied ceramics depending on the concentration of the dopant.

Figure 3 shows X-ray diffraction patterns of the studied samples of ceramics synthesized with different dopant concentrations. In the case of dopant-free ceramics, the nature of the position of diffraction reflections, as well as the ratio of intensities, indicates that the synthesized ceramics are Li_2_TiO_3_ structures with a monoclinic type of crystal lattice and space group C12/c1(15). At the same time, the ratios of the areas of reflections and background radiation were analyzed, making it possible to determine the crystal structure perfection degree; it was found that the synthesized ceramics have a high structural ordering degree of more than 90%, and their crystal structures had low porosity.

This value, as well as the phase composition of the ceramics, indicates that the chosen synthesis conditions make it possible to obtain highly ordered single-phase Li_2_TiO_3_ ceramics, which, according to scanning electron microscopy data, have a nanosized grain structure, with grain sizes of no more than 50–70 nm. Such small grains contribute to an increase in the specific surface area of ceramics, as well as having the possible effect of nanostructural ordering associated with nanosized grains and their packing density. 

According to the X-ray diffraction data, the addition of nickel to the ceramic composition in a molar ratio of 0.10 mol leads to the formation of an Li_0.94_Ni_1.04_Ti_2.07_O_7_-type substitutional solution phase with a hexagonal crystal lattice and parameters a = 5.0323 Å, c = 32.4269 Å. At the same time, the Li_2_TiO_3_ monoclinic phase with parameters a = 5.0429 Å, b = 8.8196 Å, c = 9.6813 Å and β = 99.577⁰ remains the dominant phase in the synthesized ceramics. In this case, the difference between the crystal lattice parameters and the data obtained for samples undoped with nickel oxide indicates that nickel can partially replace Li or Ti ions in the Li_2_TiO_3_ crystal lattice structure, which also leads to the formation of a substitutional solid solution phase. The ratio of the Li_0.94_Ni_1.04_Ti_2.07_O_7_ and Li_2_TiO_3_ phases is 1:4, according to the estimates of the reflection area contributions for each observed phase. In this case, the formation of a phase characteristic of a substitutional solid solution also leads to an increase in the crystallinity degree from 91% to 93.5%, which indicates an increase in structural ordering during synthesis and subsequent thermal sintering. 

According to the X-ray diffraction data, an increase in the dopant concentration to 0.25 mol leads to the displacement of the Li_2_TiO_3_ phase and the formation of a Li_0.94_Ni_1.04_Ti_2.07_O_7_ phase with parameters a = 5.0254 Å and c = 32.3188 Å. The crystallinity degree for the samples was more than 94%. In this case, according to the estimation of the crystallite size, a slight decrease in size is observed.

Thus, analyzing the data from the X-ray phase analysis, we can conclude that an increase in the concentration of the NiO dopant, and subsequent technological operations associated with the thermal sintering of the obtained samples, leads first to the formation of two-phase ceramics, and then to single-phase ceramics, with a characteristic substitutional solid solution phase of the Li_0.94_Ni_1.04_Ti_2.07_O_7_ type.

Table 1 presents the results of the crystal lattice parameters, as well as the phase ratios established by analyzing the obtained X-ray diffraction patterns. To determine the phase content, the Rietveld method was used, along with calculation Formula (1):

(1) $$V_{\mathrm{admixture}}=\frac{RI_{\mathrm{phase}}}{I_{\mathrm{admixture}}+RI_{phase}}$$

*I_phase_* is the average integrated intensity of the main phase of the diffraction line, *I_admixture_* is the average integrated intensity of the additional phase, and *R* is the structural coefficient equal to 1.45.

**Figure 4:**
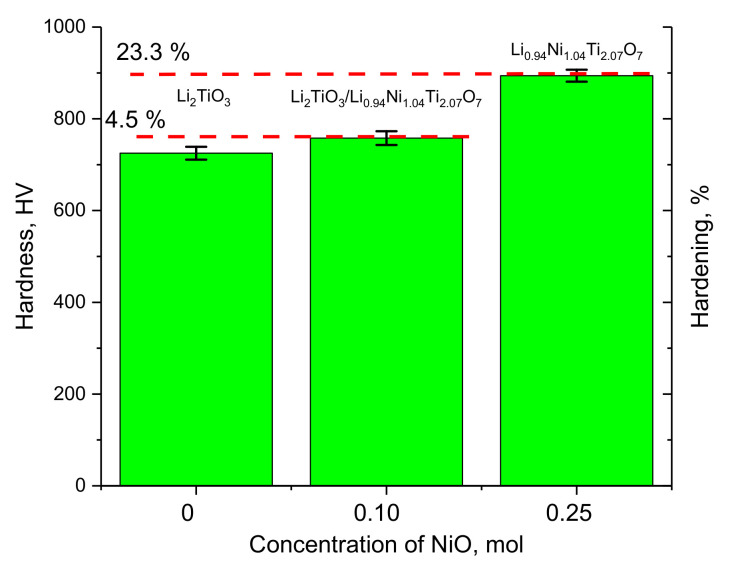
Diagram of the dependence of the change in the hardness of the studied ceramic on the concentration of the doping impurity. (Dashed lines show the values of the change in the hardening of the ceramic upon the addition of a dopant).

Figure 4 shows the results of changes in the hardness and hardening of ceramics depending on the dopant concentration. The hardening of the ceramics was calculated for the doped samples based on the ratio of the hardness value and its changes. 

As can be seen from the data presented, the formation of the Li_0.94_Ni_1.04_Ti_2.07_O_7_ phase in the structure of lithium-containing ceramics leads to an increase in hardness of 4.5%, which may be due to the hardening effect associated with the formation of additional grain boundaries and interfacial boundaries. According to the SEM image data, the addition of nanoparticles to the composition of NiO ceramics leads to a decrease in the grain size, as well as a change in shape, and the formation of dendrite-like structures. Reducing the grain size leads to a hardening effect. 

An increase in the dopant concentration from 0.10 mol to 0.25 mol, which leads to the dominance of the Li_0.94_Ni_1.04_Ti_2.07_O_7_ phase in the structure, results into the strengthening of ceramics by more than 20%. Such a change in the strength properties can also be attributed to the effect associated with dislocation hardening. An analysis of the change in the dislocation density indicates that a decrease in the grain size from 70–80 nm to 55–60 nm and 40–45 nm for dopant concentrations of 0.10 and 0.25 mol, respectively, leads to an increase in the dislocation density by factors of 1.8 and 3.2, respectively. At the same time, analyzing the data on changes in strength and hardness in the case of doped ceramics, it can be seen that the presence of interfacial boundaries has a smaller effect on the strengthening of ceramics, while an increase in dislocation density has a more pronounced strengthening effect.

**Figure 5:**
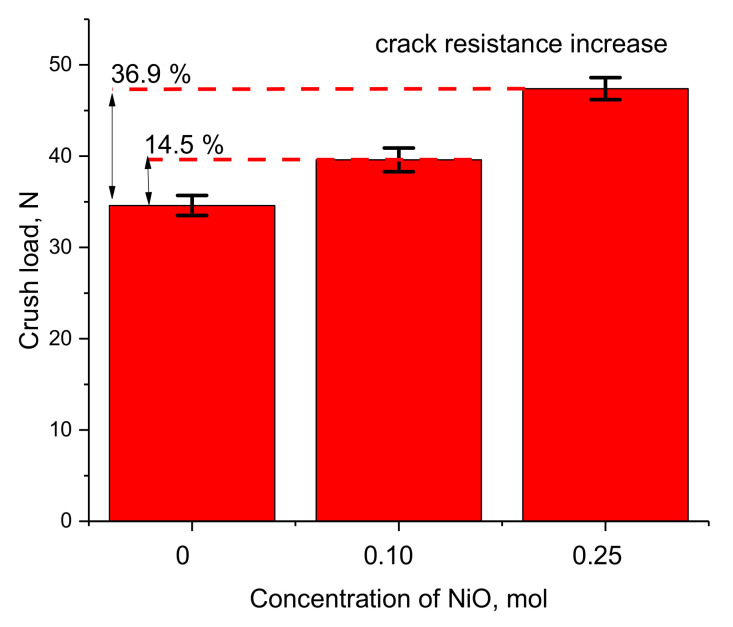
Diagram of the dependence of the change in the value of crack resistance on the concentration of the dopant in the analysis of resistance to cracking.

In addition to the change in the phase composition of ceramics on hardening and resistance to mechanical stress, the effect of the dopant is also evidenced by the results of determining the resistance of ceramics to cracking under single compression. The results of the experiments investigating crack resistance are shown in Figure 5. 

As can be seen from the presented data, the doping and subsequent phase transformation of the Li_2_TiO_3_ → Li_0.94_Ni_1.04_Ti_2.07_O_7_ type leads to an increase in crack resistance by 14.5% for two-phase ceramics, and by 36.9% for single-phase Li_0.94_Ni_1.04_Ti_2.07_O_7_ ceramics. Such an increase in crack resistance indicates that the strength properties of ceramics significantly depend on the phase composition of ceramics; in turn, doping leads to an increase in resistance to crack formation and an increase in strength. 

**Figure 6:**
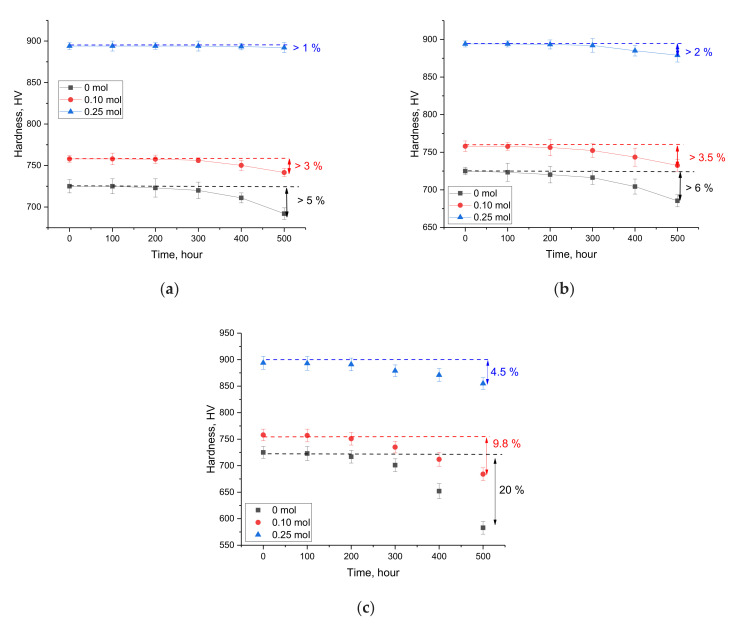
Results of the change in the hardness value of ceramics depending on time in thermal aging tests: (**a**) 500 K; (**b**) 600 K; (**c**) 700 K.

High-temperature aging tests were carried out under conditions of prolonged heating of the samples for 500 h at a temperature of 500–700 K; then, the surface resistance of the samples to mechanical stress was measured. The choice of thermal heating conditions makes it possible to simulate the performance of ceramics in a context comparable to the actual performance of reactor materials. Control measurements were carried out after every 100 h to determine the resistance to mechanical pressure and cracking under a single compression. The results of experiments related to high-temperature aging are shown in Figure 6.

The general view of changes in hardness depending on the time of testing at different temperatures indicates a deterioration in the strength of the near-surface layer of ceramics to mechanical stress. At the same time, the decrease in hardness has a pronounced dependence on both the test time and the temperature. 

In the case of an aging temperature of 500 K, a change in hardness begins to appear after 200 h of testing. At the same time, in the case of single-phase Li_0.94_Ni_1.04_Ti_2.07_O_7_ ceramics, the maximum decrease in hardness after 500 h of testing was no more than 1%, which indicates the high stability of doped ceramics. In the case of undoped Li_2_TiO_3_ ceramics and two-phase Li_2_TiO_3_/Li_0.94_Ni_1.04_Ti_2.07_O_7_ ceramics, the decreases in hardness after 500 h of testing were no more than 5% and 3%, respectively. These results indicate the ceramics’ sufficiently high resistance to aging at a temperature of 500 K. An increase in the aging temperature to 600 K leads to a slight decrease in hardness, as well as the beginning of a decrease in hardness for undoped Li_2_TiO_3_ ceramics and two-phase Li_2_TiO_3_/Li_0.94_Ni_1.04_Ti_2.07_O_7_ ceramics after 100 h of testing; this indicates an acceleration of the degradation processes. As a result of the observed changes, it can be concluded that an increase in the test temperature from 500 K to 600 K leads to an acceleration of degradation processes, leading to the destruction of crystalline and chemical bonds, as well as a decrease in strength properties. According to the analysis of the structural parameters of the studied ceramics after corrosion tests, the largest decrease in the degree of crystallinity was observed for the undoped samples; this decrease amounted to more than 15%. Such a decrease in the degree of crystallinity may be due to the occurrence of structural deformations under the influence of temperature, which subsequently leads to a softening of the near-surface layer. In the case of doped ceramics, the decrease in the degree of crystallinity was no more than 2–5%.

**Figure 7:**
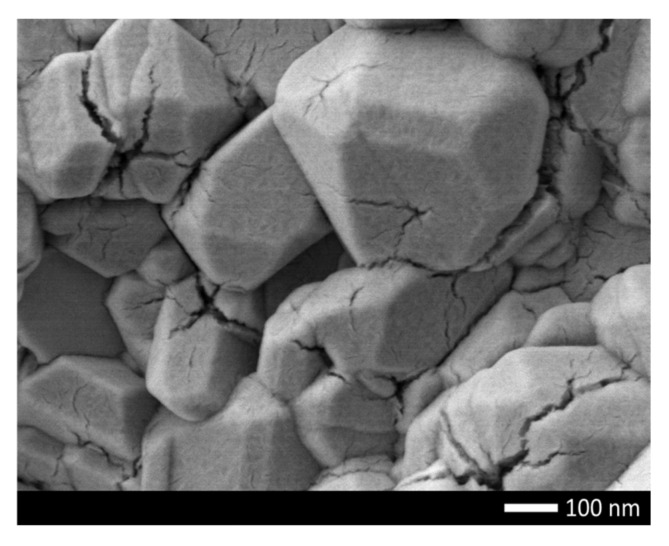
An example of the surface morphology of Li_2_TiO_3_ ceramics after 500 h of testing at a temperature of 700 K.

In the case of an aging temperature of 700 K in the case of undoped Li_2_TiO_3_ ceramics, the maximum decrease in hardness was more than 20% after 500 h of testing, which indicates a strong degradation of the strength properties of ceramics (an example is shown in Figure 7). In this case, doping and the subsequent formation of the Li_0.94_Ni_1.04_Ti_2.07_O_7_ phase leads to a more than twofold increase in the resistance of ceramics to high-temperature aging for two-phase Li_2_TiO_3_/Li_0.94_Ni_1.04_Ti_2.07_O_7_ ceramics, and a fourfold increase for single-phase Li_0.94_Ni_1.04_Ti_2.07_O_7_ ceramics.

## 4. Conclusions

The paper presents the results of a study of the effect of nickel oxide doping of lithium-containing titanates-based ceramics on the structural and strength properties of these ceramics. During the studies, it was found that the addition of NiO nanoparticles at concentrations of 0.10 mol and 0.25 mol to the composition of the initial mixtures during the synthesis of lithium-containing ceramics leads to the formation of two-phase Li_2_TiO_3_/Li_0.94_Ni_1.04_Ti_2.07_O_7_ ceramics and single-phase Li_0.94_Ni_1.04_Ti_2.07_O_7_ ceramics. At the same time, analysis of data on changes in morphological and structural features showed that the formation of the Li_0.94_Ni_1.04_Ti_2.07_O_7_ phase in the structure of ceramics leads to a change in the grain size and their reduction, and an increase in the dislocation density, which in turn has a significant effect on the strength mechanical properties. While testing the strength characteristics, it was found that doping leads to an increase in hardness and crack resistance, which is due both to a change in the phase composition of ceramics and to dislocation hardening. 

Further research in this area will aim to study the effect of doping and the formation of Li_0.94_Ni_1.04_Ti_2.07_O_7_ ceramics on radiation embrittlement during implanted helium accumulation, as well as on the processes of tritium release and the accompanying corrosion processes.

## Figures and Tables

**Table 1:** The results of changing the parameters of the crystal lattice and the ratio of the phase composition of ceramics, depending on the concentration of the dopant.

	Lattice Parameter, Å		
	Pristine	0.10 mol	0.25 mol
Li_2_TiO_3_	a=5.0637 ± 0.0027, b = 8.7663 ± 0.0035, c = 9.6984 ± 0.0024, β = 99.792°	a = 5.0429 ± 0.0015, b = 8.8196 ± 0.0025, c = 9.6813 ± 0.0031, β = 99.577°	-
Li_0.94_Ni_1.04_Ti_2.07_O_7_	-	a = 5.0323 ± 0.0026, c = 32.4269 ± 0.0032	a = 5.0254 ± 0.0012, c = 32.3188 ± 0.0034
	Phase concentration, %		
Li_2_TiO_3_	100	79.5 ± 2.2	-
Li_0.94_Ni_1.04_Ti_2.07_O_7_	-	20.5 ± 1.2	100

## Data Availability

Not applicable.
